# Evaluation and comparison of the effects of various cognitive-behavioral therapy methods on climacteric symptoms: A systematic review study

**DOI:** 10.4274/jtgga.galenos.2019.2018.0170

**Published:** 2019-08-28

**Authors:** Leila Mollaahmadi, Afsaneh Keramat, Nasrin Changizi, Mansoureh Yazdkhasti, Bahare Afshar

**Affiliations:** 1 Student Research Committee, School of Nursing and Midwifery, Shahroud University of Medical Sciences, Shahroud, Iran; 2 Reproductive Studies and Women’s Health Research Center, Shahroud University of Medical Sciences, Shahroud, Iran; 3 Ministry of Health and Medical Education, Tehran, Iran; 4 Department of Midwifery, School of Medicine, Social Determinants of Health Research Center, Alborz University of Medical Sciences, Karaj, Iran; 5 Student Research Committee, School of Nursing and Midwifery, Iran University of Medical Sciences, Tehran, Iran

**Keywords:** Climacteric, cognitive behavioral therapy, menopause, symptoms

## Abstract

**Objective:**

Climacteric syndrome, which is related to many symptoms, often causes discomfort in women. Non-pharmacologic treatment is one of the treatment options for affected individuals, and this syndrome can be cured with psychological treatments such as cognitive behavioral therapy (CBT). The present study aimed to compare the efficacy of various CBT methods on the improvement of climacteric symptoms.

**Material and Methods:**

PubMed, Scopus, Cochrane, Medline, PsycINFO, and Google Scholar were searched for relevant articles published between January 1990 and August 2018. Data extraction and quality assessment were conducted by two authors.

**Results:**

A total of 15 articles including 910 women were entered. We divided the CBT methods into two categories, face-to-face (individual and group CBT) and indirect (self-help CBT) methods. Among the three CBT approaches, three articles covered individual CBT, nine articles carried out group CBT, and in five articles, the self-help approach was used. The climacteric symptoms that improved with CBT were categorized into three groups as vasomotor symptoms, psychological symptoms, and organic disorders. Generally, the face-to-face method played a key positive effect on symptom improvement, and the group CBT approach was more effective on psychological symptoms.

**Conclusion:**

Although the indirect method is more cost-effective, it has less impact than the face-to-face method; it is better to use face-to-face approaches to achieve better results, if possible. Further studies are required in this regard, particularly in the individual and self-help CBT approaches, to measure the impact of these approaches on more varied symptoms of menopause.

## Introduction

Climacteric and menopause are closely related concepts; however, they do not denote to exactly the same thing. Climacteric is the process of aging in women, including three periods. The first stage is peri-menopause, occurring within one and eight years before the beginning of menopause. A series of gradual changes occur during this period. The second period is menopause, which is confirmed by having experienced a year of amenorrhea, and the postmenopausal stage, which is the third phase, begins when menopause is confirmed and lasts until old age (1). From a practical point of view, the term *menopause* globally refers to the aging process of the ovary and includes any period of peri-menopausal and postmenopausal in women (2).

The climacteric period can be associated with symptoms in four different classifications: 1- vasomotor vegetative symptoms (e.g. hot flashes, night sweats, palpitations); 2- psychological symptoms (e.g. anxiety, depression, nervousness, insomnia, decreased libido, memory loss, melancholy, fatigue); 3- organic disorders (e.g. osteoporosis, cutaneous atrophy, urogenital atrophy, arthralgia, myalgia); and 4- metabolic disorders (e.g. obesity, arterial hypertension) (Table 1). The pathogenesis is related to a decline in sex hormone concentration, particularly the decrease in estrogens (3,4). Moreover, some factors such as genetic and lifestyle factors, psychological disposition and personal attitudes as well as educational background (5), have a key impact in experiencing menopause in the climacteric period in women (6). The average age of menopause is 51 years (7), and the age of menopause remains constant in spite of the increased life expectancy in women, Therefore, with an increase in life expectancy, women spend about one-third of their lives after menopause and have problems caused by menopausal symptoms (8). As expressed by women, they consider menopause “the beginning of new phase of life”, “dissatisfaction with sexual acts” and “change in physical and mental health” (9). Thus, performing therapeutic interventions is essential to reduce the negative effect of climacteric syndrome on lifestyle.

Hormone replacement therapy (HRT) is the most extensively used treatment for the main symptoms of menopause, causing a 70-90% reduction of the symptoms (10). Although HRT has been the treatment of choice for climacteric syndrome for many years, uncertainty about its benefits and costs has emerged since the publication of the Women’s Health Initiative’s results (11). Many women prefer non-medical treatments for menopausal symptoms (12) and they are always concerned about the adverse effects and possible long-term health risks of HRT (13). Strong and convincing evidence exists indicating that the long-term risk of using estrogen and progestin to avoid postmenopausal diseases is much greater than its benefits (11). These results have challenged health providers to find alternative treatments for menopausal women (14). The evidence base for non-medical treatments is being increasingly examined with mixed results (4,13-22.) In addition, there has been considerable interest in developing effective nonmedical interventions to help women manage menopausal symptoms (4,17,19,23).

Considering the physical and psychological problems that occur in this period, it seems that non-medical therapies that help women to deal with their problems, particularly psychological therapies will be useful. Cognitive behavioral therapy (CBT) is one of the effective methods (24,25). Nowadays, CBT is used in the management of many conditions such as anxiety, depression, phobia, and stress (26). CBT-based psychological treatments were developed as treatments for menopausal disorders (21). This therapy helps people to think differently and due to this new thinking, they can confront undesirable events with more acceptable behaviors (27,28). In recent studies, it was shown that cognitive behavioral treatment, including psychoeducation, paced breathing/relaxation, and CBT could help women to manage symptoms such as HF/NS, which was acceptable to women, showed promise in exploratory trials of individual and group CBT, and reduced the symptoms (14,17,29).

Various CBT methods (group, individual and self-help CBT) were implemented in the climacteric period in several trials on the health of women.

The present systematic review aimed to compare the efficacy of various methods of CBT on the improvement of the climacteric symptoms.

## Material and Methods

### Search strategy

The current systematic literature review was performed using electronic databases such as PubMed, Scopus, Cochrane, Medline, PsycINFO, and Google Scholar. The search was performed from January 1990 to August 2018 by using the following related keywords in titles and abstracts (women OR female) AND (menopause* OR peri-menopause OR “post menopause”) AND (climacteric treatment OR therapy OR “cognitive behavioral therapy” OR CBT OR “psychological treatment symptom”) AND (“hot flashes” OR sweat OR anxiety OR depression OR insomnia OR “menopausal symptoms” OR “climacteric syndrome”).

Moreover, the reference section of relevant trials, systematic reviews and meta-analyses were manually checked to recognize the related trials missed by electronic database searches.

Two authors independently conducted the search and screened studies against the inclusion criteria; first, the authors independently extracted data and then checked the extracted data. Any discrepancies were resolved via discussion and consensus.

The following data were extracted with the use of PICOS criteria: population (e.g. sample size, women with natural menopause), intervention (e.g. various CBT methods: group, individual and self-help CBT, duration, length of program), comparison (e.g. non-CBT therapy group or no treatment control), outcomes (e.g. reported in the form of the improvement scores of climacteric symptoms), study design (e.g. RCT, clinical trial, quasi-experimental). Thus, the data were extracted and classified under the following headings in systematic tables (Table 1, 2, 3, 4): author, country, year (to establish a historical timeline), study design, sample size, specifications of population, comparison condition, scale, intervention, and the main findings of the studies, which can be reported in the form of scores and changes.

### Inclusion and exclusion criteria

The inclusion criteria for entering evidence in the current systematic review included original and quantitative interventional studies in English or at least with an English abstract, which could offer adequate information regarding the impact of any kind of CBT methods on the improvement of menopausal symptoms, which were published in peer-reviewed journals. Studies with randomized-control trial, clinical trial, experimental, semi-experimental, and pilot designs were entered and the subjects of the studies were healthy women in the climacteric period with normal menopause (not because of surgery) and receiving CBT for the treatment of the symptoms.

The exclusion criteria included the qualitative and quantitative interventional studies without numerical outcome data, and observational, cohort, case-control, cross-sectional, retrospective, and prospective studies were also excluded.

### Screening

A total number of 1628 articles were identified and imported to Endnote X8, and after removal of duplicates (n=415), we screened titles and abstracts of the remaining articles (n=1213). After evaluating the inclusion criteria in remaining papers, the texts of 59 potentially relevant articles were fully assessed for more screening. These articles were evaluated for eligibility, and finally, 15 studies were entered in the current systematic review.

Based on the type of CBT interventions, the entered studies were classified into two groups based on the type of CBT interventions; the first classification was face-to-face CBT, including individual and group CBT, and the second was indirect CBT, containing self-help CBT. In the indirect method, the support is provided by a professional therapist by telephone, email, or any other communication tools.

### Quality assessment

The quality of the studies was evaluated using the Cochrane Collaboration’s tool to assess the risk of bias in randomized trials by two authors independently (27). In addition, the tool has six criteria assessed in the entered studies, which are random sequence generation, allocation concealment, description of drop-outs, blinding of participants and personnel, power analysis, and intention-to-treat analysis or no drop-outs. One point was given for each criterion observed in each study. Based on this assessment tool, the quality of a study was evaluated as “high” when five or six criteria were observed, “moderate” when three or four criteria were observed, and “low” when fewer than three criteria were observed. Any disagreements between the two authors were discussed until consensus was reached and if any variation remained, it was settled through discussions with a third researcher.

## Results

**Figure 1:**
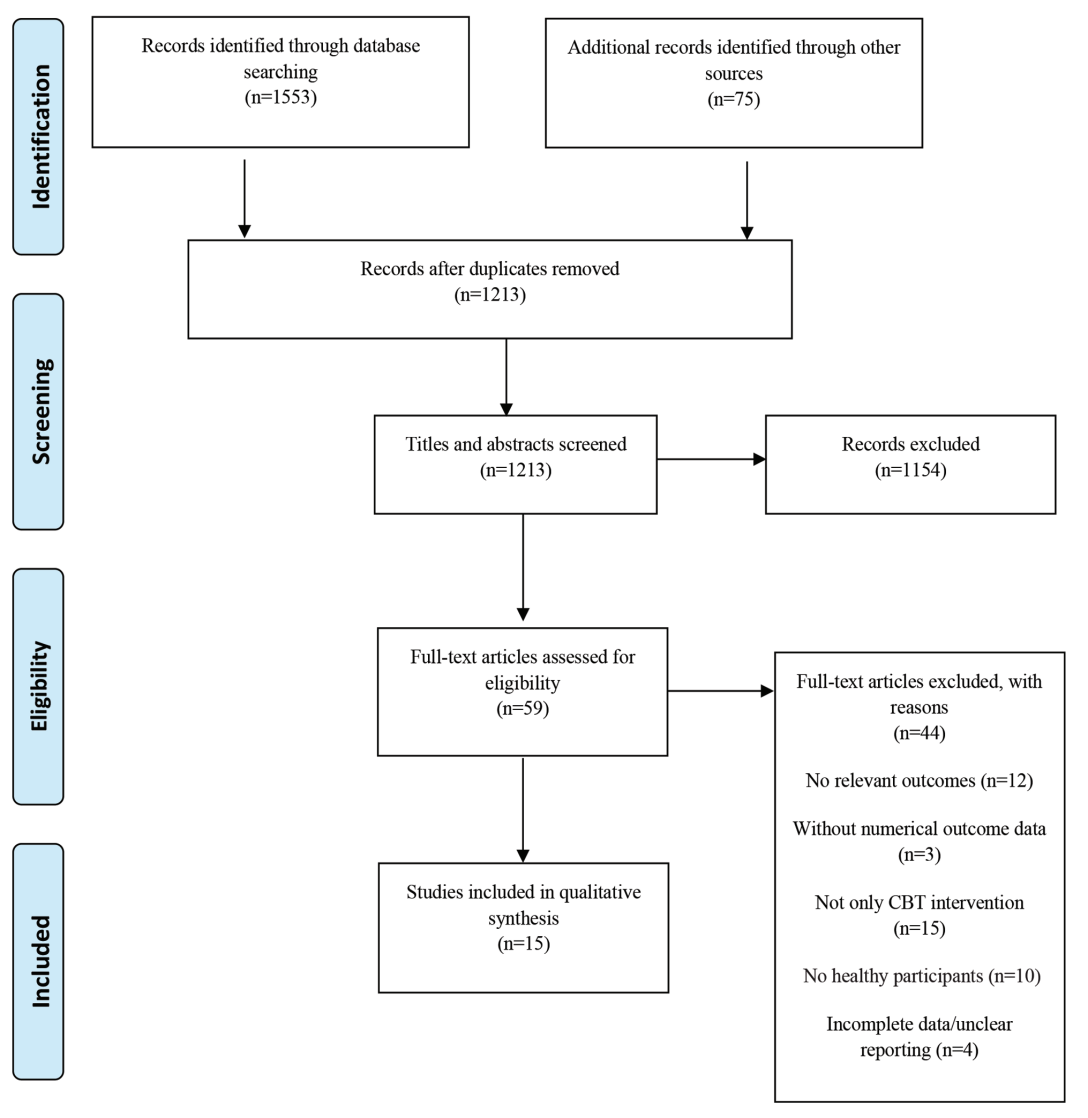
PRISMA flow diagram

From all the related papers, based on the title and abstract screening, we can observe the inclusion criteria in 15 studies. Figure 1 represents a flow diagram of PRISMA.

### Characteristics of the included studies

A total of 15 articles were published between 1996 and 2018. Among all the final articles, the designs of most studies (n=8) were randomized controlled trials (RCTs) (17,19,20,21,31,32,33,34,35), three were pilot studies (4,14,34), one of the remaining articles had a randomized clinical trial design (36), and two studies were clinical trials (33,35), we also have a quasi-experimental design in all the articles (23). Among the articles, two articles of Hassan (31) and Khoshbooii (32) were obtained from the findings of one study and had similar results.

### Demographic characteristics of subjects

According to the total number of subjects in all entered studies, 910 women were entered in the current systematic review. The sample size of the study population per study varied from 8 to 140 women, and the age range of the participants in the articles was assessed from 35 to 71 years.

The women involved in these studies were fairly healthy, mostly married or cohabiting, and had at least one child. Educational level was divided between those educated up to lower than primary school education, and the majority had at least elementary education and housekeeping (4,17,19,21,34,35,37). All of the participants were employed in one study (36). In two studies, the demographic variables were not described completely (14,33).

### Methods of recruitment

Six studies recruited participants from health centers (17,21,31,32,33,35), three through Women’s Health Clinics (4,23,34), and five studies through general practices, breast screening clinics, menopause websites, and local newspaper advertisements (14,19,20,37,38), and finally, one study recruited participants from public and private sectors (36).

The following scales were used in the entered studies to assess the symptoms changes: Insomnia Severity index, BDI-II Questionnaire, Women’s Health Questionnaire, the Depression, Anxiety, and Stress Scale-21, Blatt’s Kupperman Menopausal index, Hospital Anxiety and Depression scale, HF/NS problem-rating, Center for Epidemiologic Studies Depression scale, the Greene Climacteric scale, the Montgomery-Asberg Depression Rating scale, the Hamilton Anxiety scale, Menopause Rating scale, and the Hot Flashes Related Daily Interference scale.

The number of studies based on their countries included five studies from the United Kingdom, four from the United States, three from Iran, two from Spain, and one study from Switzerland.

### Quality assessment

In total, the six quality criteria were assessed for 15 studies. The lowest score was 1 (four studies), and the highest score was 5 (three studies). The overall study quality was low, one study (6%) was rated with a high quality, six (40%) with a moderate quality, and eight (54%) with a low quality. The descriptions of the method were as follows: generation of the allocation sequence (sequence generation) was reported in zero studies; concealment of the allocation sequence (allocation concealment) was reported in 10 studies; blinding of the main outcome assessment was described in only five studies; in 10 studies, description of drop-outs was observed; a power-analysis was conducted in nine studies, and four studies had no drop-outs.

### Features of CBT sessions

Generally, in these articles, the CBT sessions were held to improve the following climacteric symptoms, which from the highest to the lowest level, were as follows: hot flashes and night sweats (HF/NS), depression, anxiety, insomnia, nervousness, melancholy, myalgia, vertigo, fatigue, irritability, headaches, palpitations, paresthesia, dysesthesia, sleeping problems, cardiac symptoms, sexual problems, urinary symptoms, vaginal dryness, and joint and muscle pain. Table 1 presents the classification of these symptoms.

As mentioned earlier, in general, we divided the studies into two general classifications in terms of the CBT method used (face-to-face and indirect), where the face-to-face method includes individual CBT and group CBT. Based on the studies reporting the individual CBT, this approach was conducted in the form of 4-6 sessions of one hour per 6-8 weeks. In general, group CBT sessions consisted of 4 to 16 sessions of 60 to 160 minutes, usually held weekly, and women were in groups of 4 to 12 people. All studies considering the self-help CBT as a subset of indirect CBT used a booklet and participants had to complete this protocol during a 4-week period, and two studies, in addition to the booklet, had 2-week telephone guide sessions.

### Statistical analysis

To assess the effect of CBT methods on climacteric symptoms and to assess clinically meaningful individual change in symptoms, symptom changes scores were calculated as follows (mean difference):

 MD= Pre-treatment symptom score — Last post treatment symptom score 

For better a comparison between all the main results, and so as to not equalize the score before the treatment in the studies, we converted the MD score to a percentage.

Accordingly, the number in the table in percentage form represents the decrease or increase in the severity of the symptoms after the treatment (compared with the initial score).

$Percentage\;=\;\frac{MD}{Pre-treatment\;symptom\;score}$

## The Effect of CBT Methods on Climacteric Symptoms

### a. The effect of evaluating each CBT approach on symptoms reviewed in studies (Table 2, 3, 4)

### HF/NS frequency

According to the findings;

Individual CBT was able to decrease the pre-test score of HF/NS frequency up to 59% (Table 2).

Group CBT was successful in decreasing the initial score of HF/NS frequency by 3.9-40% (Table 3).

Self-help CBT made a decline in the baseline score of HF/NS frequency by 3.9-48% (Table 4).

### HF/NS problem rating

Individual CBT caused a 33% reduction from the baseline score of HF/NS problem rating (Table 2).

Group CBT was able to make a 22-52% reduction in the pre-test score of HF/NS problem rating (Table 3).

Self-help CBT was successful in decreasing the initial score of HF/NS problem-rating by 20-52% (Table 4).

### Hot flashes

Group CBT reduced baseline score of hot flashes by 11-57%.

### Night sweats

Group CBT was not able to significantly reduce night sweats. In the study by Kefeer and Blanchard (14), group CBT reduced night sweats up to 41% in the immediate group, but the score was nearly doubled in the delay group (Table 3).

### Depression

Individual CBT was able to make a 50-63% reduction in the pre-test score of depression (Table 2).

Group CBT was successful in decreasing the initial score of depression by 27-72% (Table 3).

### Anxiety

Group CBT was able to reduce baseline scores of anxiety by 18-71% (Table 3).

### Insomnia

Individual CBT caused a 73% reduction from the baseline score of insomnia (Table 2).

Group CBT could not only make a considerable failure in the baseline score of insomnia, but also caused a 19% increase in the pre-test score (Table 3).

Self-help CBT was successful in decreasing the initial score of insomnia by 71% (Table 4).

### Nervousness

Group CBT was able to make an approximately 18% reduction in the pre-test score of nervousness in women (Table 3).

### Melancholy

Group CBT was successful in decreasing the initial score of melancholy up to 41%.

### Cardiac symptoms

Group CBT could cause a 42% reduction from the baseline score of cardiac symptoms.

### Sexual problems

Group CBT was able to make a 29% reduction in the pre-test score of sexual problems.

### Vaginal dryness

Group CBT was able to reduce the pre-test score of vaginal dryness up to 29%.

### Urinary symptoms

Group CBT was not successful in decreasing the initial score of urinary symptoms and the score in the follow-up period had a 10% increase of baseline (Table 3).

### Joint and muscle pain

Group CBT was successful in decreasing the initial score of joint and muscle pain up to 16%.

### Myalgia, vertigo, fatigue, irritability, headaches, palpitations, paresthesia, and dysesthesia

Group CBT was unable to create a considerable decline in the follow-up score of each of them, separately (Table 3).

### b. The effectiveness of the face-to-face CBT method

To evaluate this method, first of all, we will determine the impact of individual and group CBT approach according to Table 2 and Table 3 and our main findings mentioned above.

### Individual CBT

Only three studies referred to this method, and if we determine which symptoms can be improved by this approach, HF/NS, the frequency in the vasomotor cluster can be indicated. Individual CBT can have excellent effects on insomnia, which is classified in the category of psychological symptoms. The overall findings of this approach cannot be regarded because few studies have evaluated the effects of individual CBT (Table 2).

### Group CBT

Since only group therapy was conducted on each of the vasomotor symptoms separately, we can conclude that group CBT could not be successful in treating most of the vasomotor symptoms, and it just improved hot flashes and cardiac symptoms among the seven symptoms of this classification. However, it can make the HF/NS rate better than with the other approaches.

Most of the psychological symptoms (except insomnia) had a greater improvement in the group CBT approach, and only vaginal dryness in the organic disorder category could be under the effect of group CBT, and most of them did not have significantly positive changes. Generally, group CBT was more effective on psychological symptoms (Table 3).

### c. The efficiency of indirect CBT method

In this part, we examine the self-help CBT approach.

### Self-help CBT

Self-help CBT approach has improved symptoms such as HF/NS frequency and problem rating, but the individual approach is more effective. Also this approach had the same positive effect as individual therapy on insomnia (Table 4).

## Discussion

Considering the many studies conducted to improve the menopause symptoms using group CBT, we can show that in general, the treatment group has more favorable effects on psychological symptoms. However, considering the fact that apart from group therapy, other approaches have not been applied to psychological symptoms and owing to the good effect of individual and self-help CBT in depression and insomnia, group CBT cannot be absolutely chosen as the best approach (31,32). Moreover, limited studies were conducted on individual and self-help CBT and most of them focused on HF/NS frequency and problem rating in each approach. Among these, individual CBT played a further role on HF/NS frequency, which due to the limited number of studies conducted using this approach, this part of our findings obtained from the results of one study cannot be generalized (17). Obviously, it is worth mentioning that group and self-help CBT also played a positive and similar role on HF/NS frequency, which resulted from more studies (33,34,35,36,37,38).

According to three articles comparing the different approaches (19,20,32), two studies compared the effects of group and self-help CBT on HF/NS frequency and problem rating. The group CBT treatment consists of psycho-education, stress management, paced breathing, and self-help CBT includes a self-help book that is learned during a four-week course and two phone calls made by a psychologist. Both of them, as already mentioned, indicated an almost equal effect of the two approaches; however, group CBT was somewhat more successful than self-help CBT (19,20), consistent with our findings.

In the study of Khoshbooii (32), the impacts of individual and group CBT on depression were compared with each other. The individual sessions are tailored to the needs of women and are flexible, but the general format of CBT sessions covered the main components such as psycho-education, cognitive interventions, behavioral interventions, assigning homework, and relapse prevention. According to their findings, both approaches had the same effect on depression, and the effect of individual CBT was negligibly greater than group CBT (32). In addition, as mentioned earlier, both group and individual CBT had a positive and significant impact on depression but the findings from group therapy were more widespread (32,34,35), which could be a result of the alterations in the conditions of the samples, the number of treatment sessions, the content or the kind of follow-up in studies; therefore, group CBT cannot be considered a guaranteed approach, but if properly implemented, it can reduce up to 72% of the initial depression score; otherwise, it can only be up to 27% effective. Thus, the preliminary treatment approach for depression can be group CBT sessions held in good conditions.

Based on the findings of the present study, it can be concluded that if an individual has an insomnia problem, group CBT cannot produce a good result, but individual and self-help approaches can reduce over 70% of the initial insomnia score. Furthermore, in a study by Keefer and Blanchard (14) the intervention group was classified into two immediate and delayed treatment groups in the case of assessing night sweats, depression, and total vasomotor symptoms. Treatment sessions were designed weekly and consist of education, relaxation training, and cognitive restructuring. In this regard, they reported a positive effect in the group with immediate treatment, but in the group whose treatment was delayed, the result was the opposite, and all of these three scores were increased. For example, the score for night sweats was more than twice the initial score. According to this finding, the start time of group therapy is noticeable, and if the treatment begins at a later stage, the result can be obtained in the opposite way (14).

Although in the study of Larroy García and Gómez-Calcerrada (4), the symptoms measured by the Kupperman and Blatt Menopausal index questionnaire separately did not have a significant alteration after group CBT, the total score represents a 20% decrease from the initial score, indicating the effectiveness of the group approach.

### Study limitation

We were not able to perform a meta-analysis in the present study due to the alteration in the questionnaires used to measure the symptoms, and the difference in the implementation method, including the number of treatment sessions or the number of participants in the group meetings. Moreover, as a result of the low and moderate quality of most studies involved in this systematic review, more studies with high quality should be conducted in individual and self-help CBT approaches to measure the impact of these approaches on more varied symptoms of menopause.

It can be concluded that although the indirect method is more cost-effective, it has less impact than the face-to-face method, and if there are possibilities, it is better to use face-to-face approaches to achieve a better result. However, in countries with less facilities, self-help CBT (indirect methods) can be beneficial.

## Figures and Tables

**Table:**
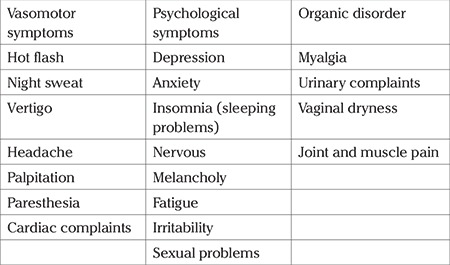
Table 1 The classification of the climacteric symptoms improved by CBT

**Face-to-Face CBT methods:**
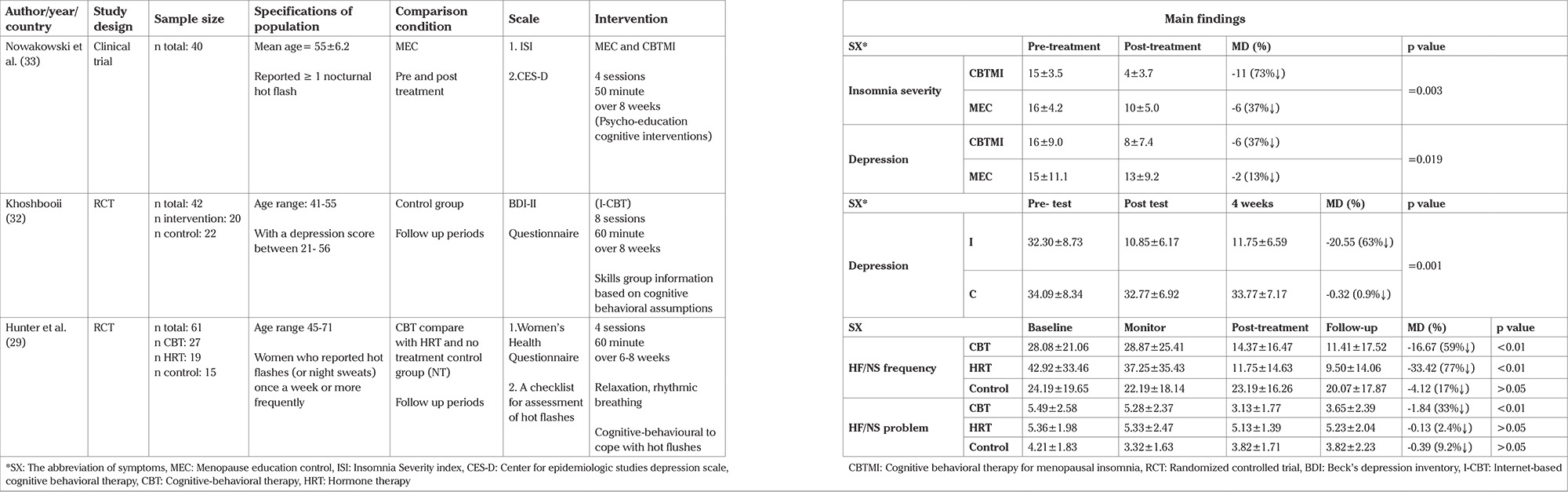
Table 2 The efficiency of individual CBT on Climacteric symptoms

**Table:**
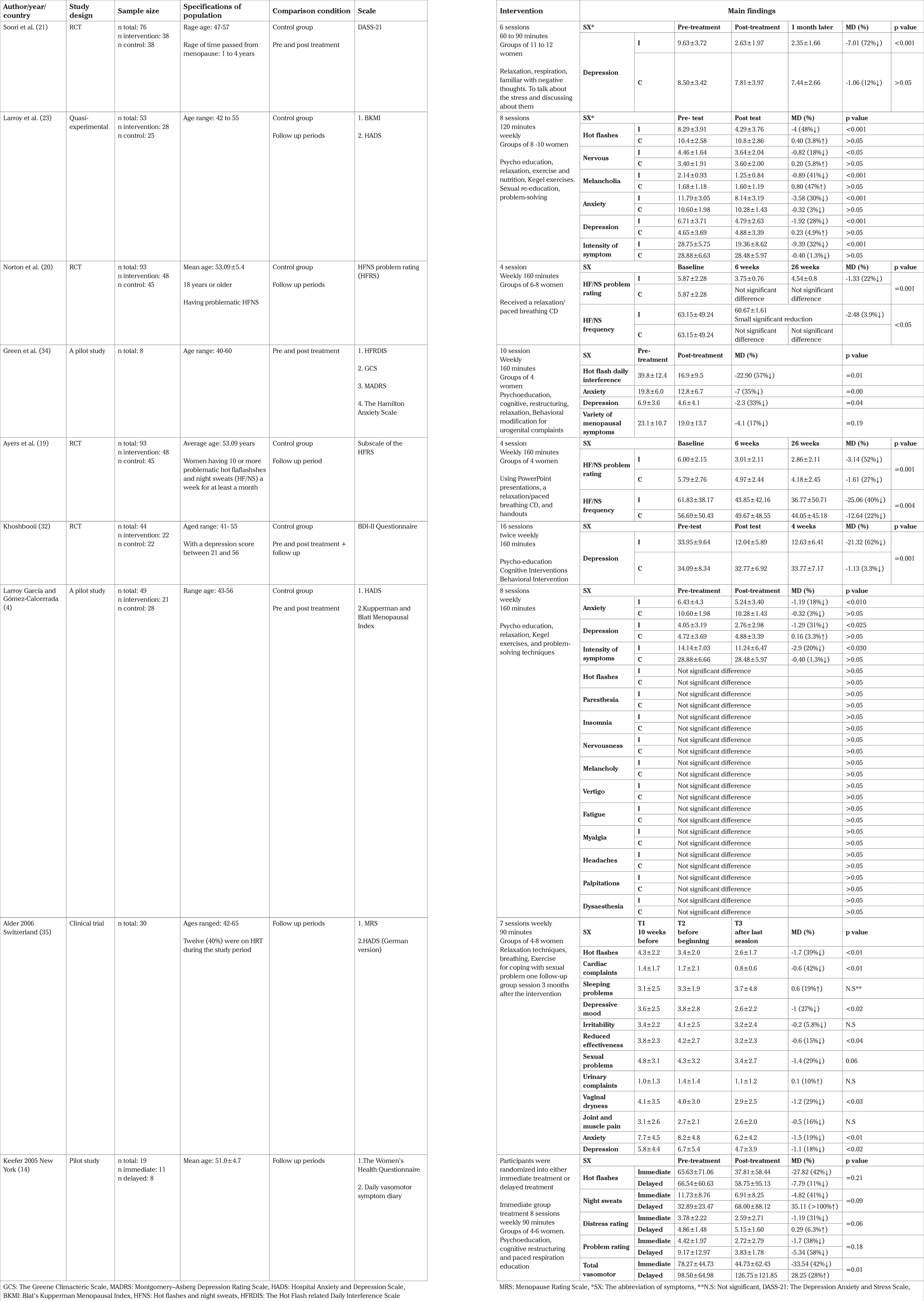
Table 3 The efficiency of group CBT on menopausal symptoms

**Indirect CBT methods:**
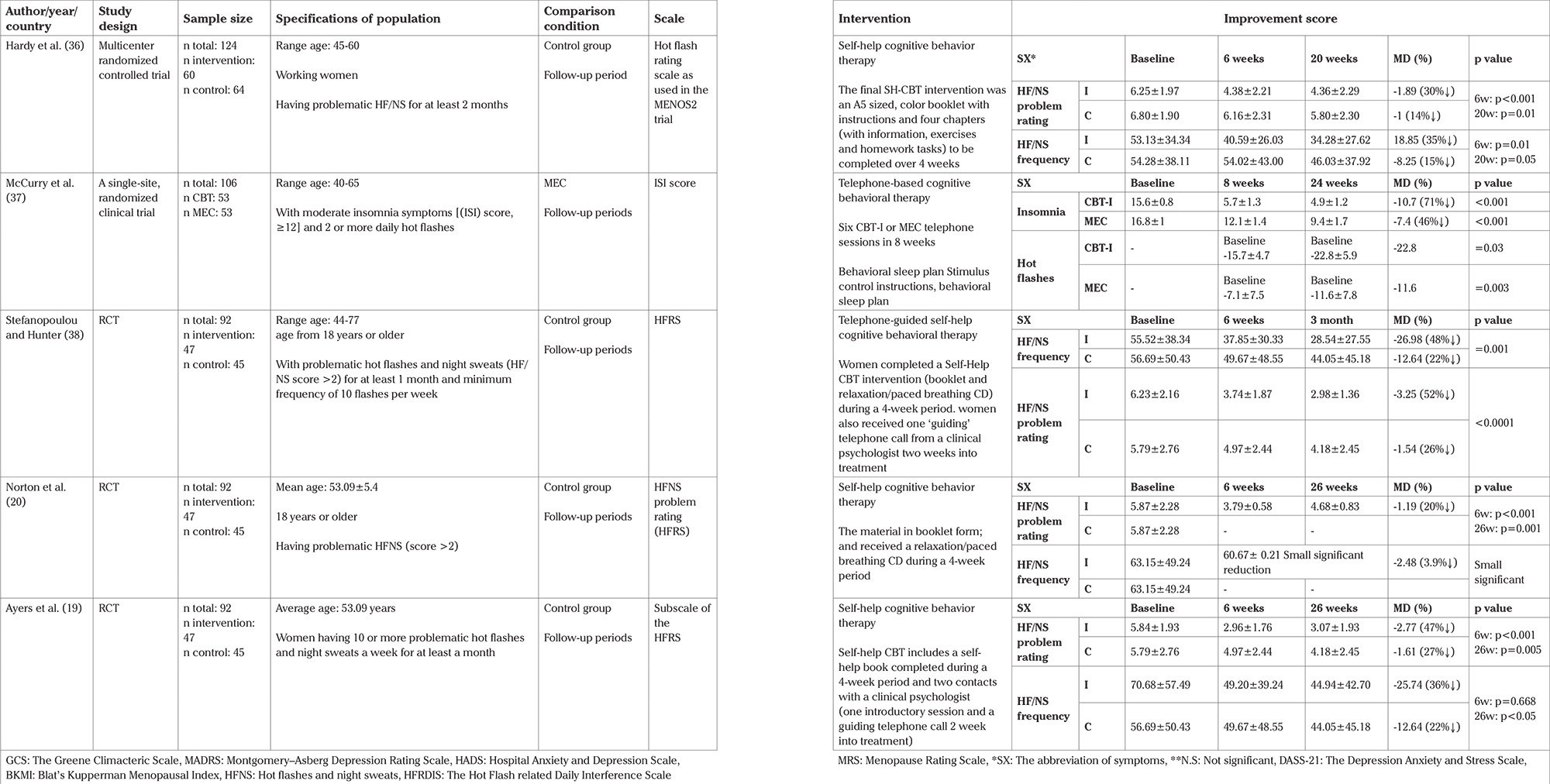
Table 4 The efficiency of self-help CBT on menopausal symptoms
